# The Relationship Between Food Consumption and Bowel Symptoms Among Patients With Rectal Cancer After Sphincter-Saving Surgery

**DOI:** 10.3389/fmed.2021.642574

**Published:** 2021-06-21

**Authors:** Wen Liu, Jian Min Xu, Yu Xia Zhang, Hui Juan Lu, Hai Ou Xia

**Affiliations:** ^1^School of Nursing, Fudan University, Shanghai, China; ^2^Shanghai University of Medicine and Health Sciences, Shanghai, China; ^3^Department of General Surgery, Zhongshan Hospital, Fudan University, Shanghai, China; ^4^Zhongshan Hospital, Fudan University, Shanghai, China

**Keywords:** rectal cancer, diet, food consumption, bowel symptoms, sphincter-saving surgery

## Abstract

Dietary self-management is an important strategy for controlling bowel symptoms after sphincter-saving surgery; however, the dietary factors influencing bowel symptoms are not completely clear. This study aimed to explore the relationship between the specific consumption of food components and bowel symptoms. This study applied a cross-sectional study design. Using convenience sampling, a total of 169 patients with rectal cancer after sphincter-saving surgery were selected from a tertiary hospital. Data were collected through three questionnaires, including general and treatment-related questionnaires, the Memorial Sloan Kettering Cancer Center (MSKCC) bowel function scale—Chinese version, and the Food Frequency Questionnaire. Multiple linear regression analysis was used to analyze the collected data. It was found that the consumption of fruit, cholesterol, and protein and the interaction of cereals and milk products were the main dietary factors affecting bowel symptoms in patients after sphincter-saving surgery. The consumption of protein and fruit was negatively correlated with the symptoms of frequent and urgent defecation, and the consumption of fruit and protein was negatively correlated with general bowel function. The consumption of fruit was negatively correlated with the abnormal feeling of defecation, and the interaction between cereals and milk products was positively correlated with the abnormal feeling of defecation. The results of this study provide evidence for medical staff to further develop scientific dietary education programs to relieve bowel symptoms and promote the quality of life of patients in the future. More research is also needed to explore the mechanisms of the effects of different food components on bowel symptoms in patients after sphincter-saving surgery in the future.

## Introduction

Colorectal cancer (CRC) is the third most prevalent cancer globally and is among the leading causes of mortality in the world (1). Due to changes in the lifestyle and dietary patterns of Chinese residents, the incidence of CRC has increased in recent years (2).

Rectal cancer is primarily treated with surgical removal, accompanied by chemotherapy and radiotherapy when needed (3). With the increasing development of surgical techniques in rectal cancer treatment, low anterior resection (LAR) with total mesorectal excision (TME) is now the gold standard for the treatment of non-disseminated rectal cancer, aiming at saving the sphincter of patients (4). However, saving the sphincter does not mean saving its function, and different bowel symptoms frequently emerge after sphincter-saving surgery, such as fecal incontinence, urgent and frequent defecation, laborious defecation, and bowel movements at night. These symptoms are collectively called low anterior resection syndrome (LARS) (5). Over 70% of patients after sphincter-saving surgery have experienced these varied symptoms, which severely influence the patients' quality of life (6–8). Although defecation dysfunction improves over time after surgery, many studies have found that these symptoms may still exist up to 15 years after surgery (9). Therefore, defecation dysfunction may be permanent and not temporary, and patients seldom fully recover (10).

Compared with the high prevalence of defecation dysfunction, effective therapy to control these symptoms is limited. Patients need to control bowel symptoms nearly all by themselves, and self-management has become the main method, including diet modification, bowel medication, exercise and practice management, and psychological adjustment. Dietary change has been reported to be the most common strategy among these methods, and more than 96% of patients reported a change in diet to control bowel symptoms in the study of Yin et al. (11). Dietary change plays an irreplaceable role in the management of defecation dysfunction because different dietary components may influence defecation by changing intestinal motility, moderating gut microbiota, and altering fecal morphology (12). Nikoletti et al. investigated the self-management status of patients after sphincter-saving surgery, and more than 30% of patients revealed a close relationship between bowel symptoms and dietary behaviors (13).

At present, research focusing on the dietary-influencing factors of defecation dysfunction is still scant. In past studies, the possible dietary factors of defecation dysfunction were mainly identified by qualitative research design, exploring the feelings and experiences of patients after sphincter-saving surgery. Landers et al. conducted semi-structured interviews with 143 patients after sphincter-saving surgery, and sweet foods such as chocolate were noted to aggravate diarrhea (14). Sun et al. enrolled 63 patients after sphincter-saving surgery and conducted 13 focus group interviews with the purpose of exploring the strategies applied by patients to control these bowel symptoms (15). The findings of this study revealed that some foods might cause symptoms such as diarrhea, constipation, and bloating, such as spicy foods, fatty foods, and dairy products, while other foods were good for patients' defecation, such as liquid food and high-fiber food and prune juices. Taylor et al. pointed out that one or two small changes to a person's diet can make significant differences. Foods that often result in problems by creating more frequent defecation or looser stool are caffeine, insoluble fiber from nuts, the skins and peas of many fruits and vegetables, fats, and spices (16).

According to the subjective data collected in the past studies, the effects of foods on bowel symptoms were inconsistent and individual differences existed in them. In the study of Sun et al., some food categories, including vegetables and fruits, were reported as being harmful by some patients but helpful for others. All the past studies just investigated the effects of different food categories on bowel symptoms, and none of them reported the direct relationship between specific consumption of food components and bowel symptoms of patients after sphincter-saving surgery. Among all food components, fiber is pointed out by some researchers to be related to fecal incontinence; however, patients with fecal incontinence in most of these studies were not the patients after sphincter-saving surgery. In a large sampling prospective cohort study conducted by Staller et al. (12), 58,330 old women were enrolled in a 4 year follow-up during the past 10 years. The results suggested that the rate of fecal incontinence of people taking 25 g of fiber per day was 18% lower than the rate of fecal incontinence of people taking 13.5 g of fiber per day. No studies have specially discussed on the effect of fiber consumption of food on bowel symptoms of patients after sphincter-saving surgery until now.

Above all, there are limited studies focusing on the effects of dietary factors on bowel symptoms among patients after sphincter-saving surgery. The conclusions among the limited studies are not consistent with each other. Patients mostly relied on self-management and trial and error to control bowel symptoms. There are also no studies discussing and analyzing the direct relationship between consumption of food component and bowel symptoms among patients after sphincter-saving surgery; thus, the specific effects of foods on bowel symptoms are not clear. The lack of professional instructions has become a prominent problem for self-management. Therefore, more studies are needed to explore the effect of specific dietary components on bowel symptoms to further understand the relationship between diet and defecation dysfunction.

Thus, this study aimed to investigate the direct relationship between the consumption of food components and bowel symptoms and further identify the dietary influencing factors of defecation dysfunction. Therefore, evidence could be provided from this study for clinical caregivers to design scientific dietary interventions to control the bowel symptoms of patients after sphincter-saving surgery.

## Methods

### Study Design

A cross-sectional observational design was applied in this study.

### Setting and Participants

Rectal cancer patients after sphincter-saving surgery were conveniently recruited from a tertiary hospital in East China from July to December 2019. Inclusion criteria were as follows: (1) primary rectal cancer by pathology, (2) finishing the sphincter-saving operation between 2 months and 2 years before recruitment, and (3) voluntary participation with informed consent. The exclusion criteria were as follows: (1) hearing or cognitive disorders, (2) complications (anastomotic fistula, rectovaginal fistula, etc.) after surgery, (3) having suffered from intestinal problems such as inflammatory bowel disease or irritable bowel syndrome before the surgery, and (4) incomplete medical records.

An *a priori* sample size calculation specified that at least a sample of 85 patients was necessary to detect a moderate correlation (correlation = 0_3), with a power of 80% and a level of significance of 0.05. In total, 169 participants were included in this study, and the demographic data are shown in Table 1.

**Table 1:** Participants' characteristics and differences of the bowel function in different groups (*N* = 169).

	***N* (%)**	**Mskcc-total score**			**Frequent defecation-subscale score**		
		**Mean SD**	**T (F)**	***P***	**Mean SD**	**T (F)**	***P***
**Gender**							
Male	109 (64.5)	62.78 ± 6.05	0.458	0.648	35.76 ± 4.53	0.090	0.928
Female	60 (35.5)	62.26 ± 6.06			35.67 ± 5.25		
**Age (years)**							
≤ 40	7 (4.1)	65.4 ± 6.73	2.534	0.044	36.80 ± 4.98	2.374	0.056
(40, 50)	21 (12.4)	60.07 ± 5.52			34.13 ± 4.88		
(50, 60)	53 (31.4)	63.58 ± 5.81			36.26 ± 4.45		
(60, 70)	52 (30.6)	63.78 ± 5.96			37.00 ± 4.57		
>70	36 (21.5)	60.38 ± 5.93			33.85 ± 4.93		
**Employment status**							
Employed	67 (39.7)	62.73 ± 5.34	0.198	0.844	36.02 ± 4.38	0.547	0.585
Unemployed	102 (60.3)	62.51 ± 6.48			35.53 ± 5.03		
**Timing (months)**							
(0, 3)	37 (21.9)	62.22 ± 4.97	0.227	0.877	35.22 ± 4.34	1.363	0.258
(4, 6)	31 (18.3)	62.32 ± 7.02			34.23 ± 5.28		
(7, 12)	41 (24.3)	62.24 ± 6.03			36.03 ± 4.95		
(13, 24)	60 (35.5)	63.21 ± 6.26			36.6 ± 4.58		
**Education**							
Less than junior school	37	62.27 ± 7.37	0.284	0.754	35.68 ± 5.35	0.891	0.413
Middle and high school	63	62.49 ± 5.59			35.35 ± 4.60		
College graduate and higher	21	63.48 ± 4.75			36.95 ± 4.19		
**Family economy**							
Just enough	94 (55.6)	61.70 ± 6.17	−1.734	0.085	35.16 ± 4.99	−1.4	0.164
rich	75 (45.4)	63.60 ± 5.76			36.37 ± 4.47		
Distance between anal and tumor (cm)		62.53 ± 6.02					
(0–5)	35 (20.7)	60.74 ± 5.59	3.721	0.045	34.76 ± 5.52	2.966	0.055
(5–10)	92 (54.4)	62.00 ± 6.06			35.29 ± 4.63		
(11–15)	36 (21.3)	63.54 ± 5.21			37.08 ± 4.06		
(15–20)	6 (3.6)	70.90 ± 9.81			40.25 ± 3.20		
**Radiotherapy status**							
Haven't received	91 (53.8)	63.23 ± 6.35	1.252	0.213	36.12 ± 4.76	0.983	0.328
Have received	78 (46.2)	61.87 ± 5.61			35.27 ± 4.79		
	***N*** **(%)**	**Dietary factors-subscale**			**Feeling of defecation-subscale**		
		**Mean SD**	**T (F)**	***P***	**Mean SD**	**T (F)**	***P***
**Gen der**							
Male	109 (64.5)	14.09 ± 1.92	1.922	0.057	12.94 ± 1.96	−0.837	0.404
Female	60 (35.5)	13.35 ± 2.21			13.23 ± 1.67		
**Age (years)**							
≤ 40	7 (4.1)	15.4 ± 3.13	2.453	0.050	13.20 ± 1.92	0.232	0.920
(40, 50)	21 (12.4)	13.0 ± 1.81			12.93 ± 1.53		
(50, 60)	53 (31.4)	14.26 ± 1.77			13.05 ± 1.45		
(60, 70)	52 (30.6)	13.92 ± 2.33			12.86 ± 2.34		
>70	36 (21.5)	13.23 ± 1.68			13.31 ± 1.91		
**Employment status**							
Employed	67 (39.7)	13.88 ± 1.53	0.210	0.834	12.83 ± 1.510	−0.995	0.322
Unemployed	102 (60.3)	13.79 ± 2.35			13.18 ± 2.06		
**Timing (months)**							
(0, 3)	37 (21.9)	14.19 ± 1.94	0.941	0.423	12.81 ± 1.52	3.028	0.032
(4, 6)	31 (18.3)	14.0 ± 2.29			14.09 ± 1.69		
(7, 12)	41 (24.3)	13.31 ± 2.04			12.90 ± 1.93		
(13, 18)	60 (35.5)	13.86 ± 2.01			12.74 ± 1.97		
**Education**							
Less than junior school	37	13.95 ± 2.29	0.194	0.824	12.65 ± 1.99	2.941	0.057
Middle and high school	63	13.71 ± 2.09			13.43 ± 1.73		
College graduate and higher	21	13.95 ± 1.50			12.57 ± 1.86		
**Family economy**							
Enough	94 (55.6)	13.67 ± 2.09	−0.877	0.382	12.88 ± 2.06	−1.041	0.300
Rich	75 (45.4)	14.01 ± 2.10			13.23 ± 1.62		
**Distance between anal and tumor (cm)**							
(0–5)	35 (20.7)	13.84 ± 1.63	1.659	0.181	13.44 ± 1.76	0.604	0.614
(5–10)	92 (54.4)	13.79 ± 2.12			12.92 ± 1.86		
(11–15)	36 (21.3)	13.58 ± 2.02			12.88 ± 1.66		
(15–20)	6 (3.6)	16.00 ± 2.94			13.50 ± 3.79		
**Radiotherapy status**							
Haven't received	91 (53.8)	14.22 ± 2.09	2.285	0.024	12.89 ± 1.79	−0.947	0.345
Have received	78 (46.2)	13.38 ± 2.01			13.21 ± 1.96		

### Research Tools

A multi-item questionnaire was used to collect data, including the sociodemographic information and clinical information of patients. Patients' bowel symptom experiences were measured with the Memorial Sloan Kettering Cancer Center's bowel function scale. Patients' eating situations were measured using the Food Frequency Questionnaire (FFQ). All the information was collected by trained investigators at the hospital clinic.

#### General Information Questionnaire

The general information questionnaire consisted of 10 items, including age, sex, employment status, education level, and tobacco and alcohol use.

#### Clinical Information Questionnaire

The clinical information questionnaire collected the past medical information and the data related to the treatment of rectal cancer, including history of past chronic disease (inflammatory bowel disease, diabetes, hypertension, and cardiac disease) and surgery, history of personal and family cancer, type of surgery, tumor classification, differentiation degree, length of bowel being removed, anastomotic site, lymphatic metastasis, adjunct therapy before and after surgery, and dietary supplement usage.

#### Memorial Sloan Kettering Cancer Center's Bowel Function Scale—Chinese Version

This scale was developed by Temple et al. (17) to assess the bowel function of patients after sphincter-saving surgery and includes 18 items. We applied the Chinese version of the Memorial Sloan Kettering Cancer Center (C-MSKCC) bowel function scale, consisting of three dimensions: (1) frequent and urgent defecation, (2) effects of diet on defecation, and (3) abnormal feelings of defecation. This scale adopted a five-point Likert scale ranging from “always” (1 point) to “never” (5 points), with a total score of 18 to 90 points. A higher score indicated a better bowel function. The Cronbach's coefficient of the Chinese version has been confirmed to range from 0.602 to 0.856 in past research (18). In this study, Cronbach's coefficient of the Chinese version of the MSKCC bowel function scale was 0.886.

#### Food Frequency Questionnaire

Dietary behaviors were assessed with a 100-item quantitative FFQ, reflecting the food consumption for the main food items by investigating the intake frequency of certain foods and the portion consumed every time. The FFQ was designed by the Chinese Center of Disease Control and Prevention in 2010 to monitor people's nutrition and diet. More than 100 food items were assigned to eight food categories. Intake frequency was estimated by using a scale of categories with response in “times per day,” “times per week,” “times per month,” and “times per year.” By multiplying the frequency and portion size, the average consumed amount was calculated and expressed as intake in grams per day. Energy and nutritional intake are estimated with regard to participants' frequency ratings and portion. Nutritional analysis was performed by the Nutrition Calculation Software, which was developed to calculate the intake consumption of different food categories, energy, and food nutrients.

### Statistical Analysis

SPSS 21.0 was used for the statistical analysis, and the statistical significance was set at *p* < 0.05 (two-tailed). Patients were excluded from the analysis if more than 30% of the data were missing. The results of FFQ were firstly recorded into the Nutrition Calculation Software and then the consumption of different food components were calculated by this software. Continuous variables were described using the mean and standard deviation, and categorical variables were described using frequencies and percentages. Independent-sample *t*-tests and analyses of variance (ANOVA) were conducted to analyze the differences in bowel symptoms from participants with different sociodemographic characteristics. Two-way ANOVA was used to analyze the interaction of different foods on bowel symptoms. Univariate analysis and multiple stepwise regression were used to analyze the dietary-influencing factors of different bowel symptoms.

### Ethical Considerations

Ethical approval for the study was granted by the Ethics Committee of ZhongShan Hospital, Fudan University (B2019-318R).

## Results

### Participants' General Information

Finally, 178 questionnaires were collected, and incomplete questionnaires or all entries with the same answer were excluded. A total of 169 questionnaires were accepted, and the questionnaire response efficiency was 94.9%.

The sociodemographic data and the treatment information of the participants are presented in Table 1. The participants' mean age was 60.57 years (SD = 11.396), and the majority of them were male (64.5%), aged over 60 years old (52.1%), and had poor or moderate family income (79.3%). A total of 60.3% of the participants had retired, and most participants had not entered college to obtain a bachelor's degree or higher (82.6%). The vast majority of participants experienced light labor intensity in their daily lives (86.8%). Most participants did not have the habit of smoking tobacco (59.5%) or consuming alcohol (80.2%) in the past 3 years. The length of time since surgery was from 2 to 13 months, and most participants underwent robotic anterior resection (89.3%). A total of 85.1% of them received chemotherapy, and 46.3% of them received radiotherapy after surgery until the time of this investigation. The average distance between the anal and low margins of the tumor ranged from 3 to 16 cm. The length of the bowel removed ranged from 6 to 18 cm.

### Scores of the C-MSKCC and Its Three Subscales

The mean score of the C-MSKCC was 62.60 (SD = 6.031), while the mean score of the subscale of frequency and urgency of defecation was 35.73 (SD = 4.771), the mean score of the subscale of effects of diet on defecation was 13.839 (SD = 2.052), and the mean score of the subscale of abnormal feeling of defecation was 13.04 (SD = 1.864). The means and standard deviations of the Chinese version of the MSKCC bowel function scale and the three subscales are shown in Table 2.

**Table 2:** Scores of C-MSKCC and its three subscales.

	**Mean ± SD**	**Entry**	**Score/item**
Mskcc-questionnaire total score	62.60 ± 6.031	18	3.48
Frequency-subscale	35.73 ± 4.771	10	3.57
Dietary factors-subscale	13.83 ± 2.052	4	3.46
Feeling of defecation-subscale	13.04 ± 1.864	4	3.26

### Analysis of General and Clinical Factors Influencing Bowel Function

The results showed significant differences (*p* < 0.05) in age, distance between anal tissue and the tumor, timing after surgery, and radiotherapy status, which are shown in Table 1.

### Two-way ANOVA of Interaction Effect Between Different Foods on Bowel Symptoms

Two-way ANOVA was conducted to explore the effect of interaction among different types of foods on bowel symptoms. After adjustment for other confounding factors, two-way ANOVA showed a significant interaction effect between milk products and fruits for the general bowel function (*p* = 0.02), the interaction effect between fruits and milk products was significant for abnormal feeling of defecation (*p* = 0.047), the interaction effect between cereals and milk products was significant for abnormal feeling of defecation (*p* = 0.002), the interaction effect between nuts and beans and milk products was significant for abnormal feeling of defecation (*p* = 0.017), and the interaction effect between livestock and poultry meat and nuts and beans was significant for abnormal feeling of defecation (*p* = 0.041). The interaction effect between different foods for the frequent and urgent defecation and for the effects of diet on defecation was not significant (*p* > 0.05) (Table 3).

**Table 3:** Two-way ANOVA of interaction effect between different foods on bowel symptoms.

	***F***	***P***	***R^**2**^***	***Adjusted R^**2**^***
**General bowel function**				
Fruits	7.671	<0.001	0.318	0.206
Milk products	0.048	0.986		
Fruits*milk products	2.419	0.020		
**Abnormal feeling of defecation**				
Cereals	2.616	0.055	0.319	0.207
Milk products	2.452	0.068		
Cereals*Milk products	3.463	0.002		
**Abnormal feeling of defecation**				
Fruits	5.679	0.001	0.276	0.148
Milk products	6.825	<0.001		
Fruits*Milk products	2.054	0.047		
**Abnormal feeling of defecation**				
Nuts and beans	0.581	0.629	0.258	0.127
Milk products	3.445	0.020		
Nuts and beans*Milk products	2.473	0.017		
**Abnormal feeling of defecation**				
Livestock and poultry meats	0.885	0.451	0.213	0.065
Nuts and beans	0.265	0.850		
Livestock and poultry meats*nuts and beans	2.055	0.041		

*Two-way ANOVA analysis have all been conducted after the adjustment of age, status of radiotherapy, distance between the margin of anal and tumor, consumption of energy*.

### Regression Analysis of Diet Consumption on General Bowel Function

Multivariate regression analysis was conducted with the C-MSKCC score, the frequency and urgency of defecation score, the effects of diet on defecation score, and the abnormal feeling of defecation score as the dependent variables. Quantiles of food components and the interaction between foods which showed significant effect for general bowel function were entered as independent variables. After adjusting the confounding factors, the regression results showed that protein consumption (β = −1.941, *p* < 0.01) and fruit consumption (β = −1.418, *p* < 0.01) had significant main effects on the C-MSKCC score, and the regression results showed an *R*^2^ of 22.7%, indicating that protein consumption and fruit consumption could explain 22.7% of the variance in general bowel function (Table 4).

**Table 4:** Regression analysis of diet consumption on general bowel function.

**Variable**	***B***	***SE***	***t***	***p***
Constant	70.941	1.458	48.640	<0.001
Protein	−1.941	0.439	−4.425	<0.001
Fruit	−1.418	0.439	−3.233	0.002

*R^2^ = 0.24, adjusted R^2^ = 0.227, F = 18.666*.

**Figure 1:**
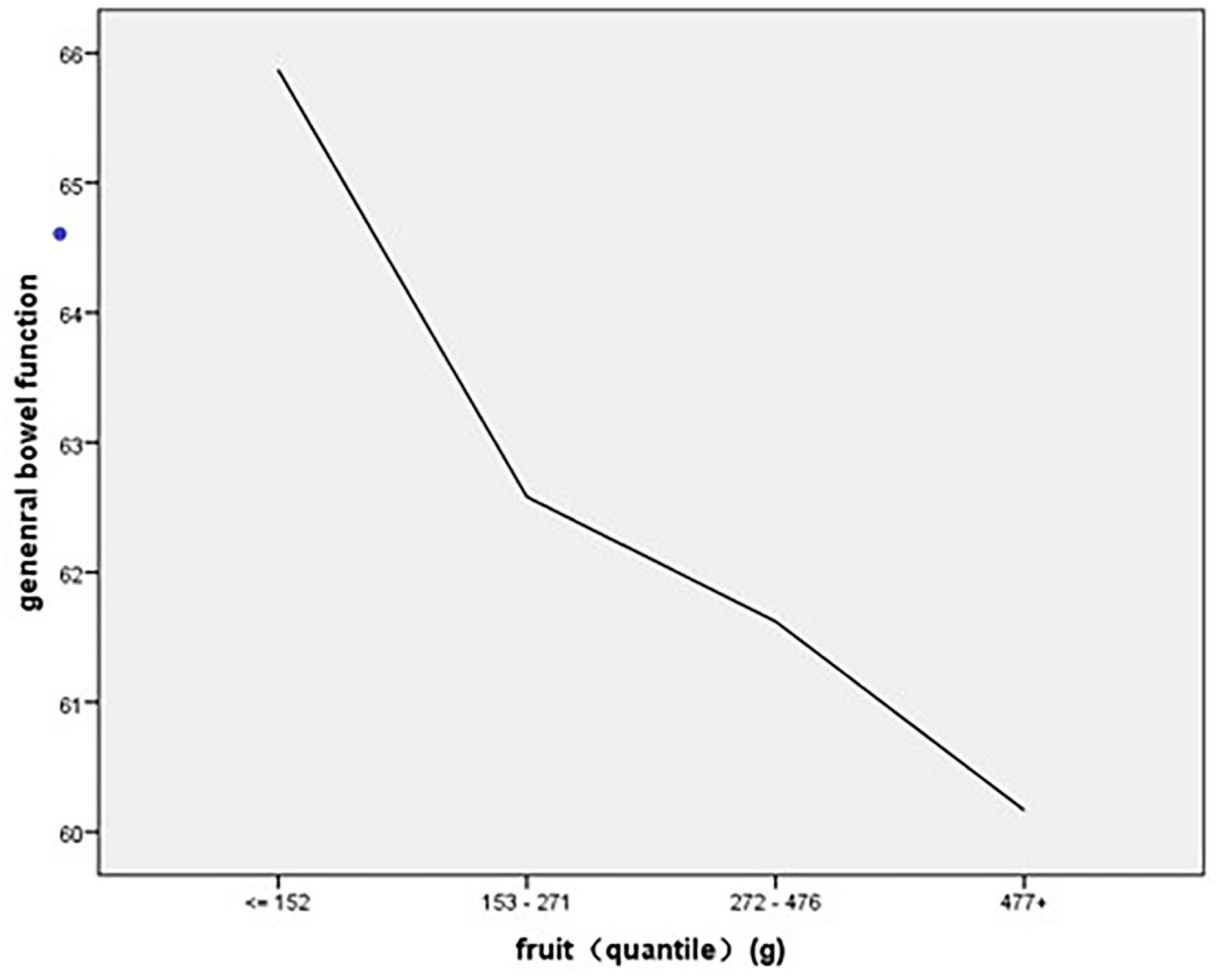
The association of stratified quintiles of consumption of fruits with general bowel function.

**Figure 2:**
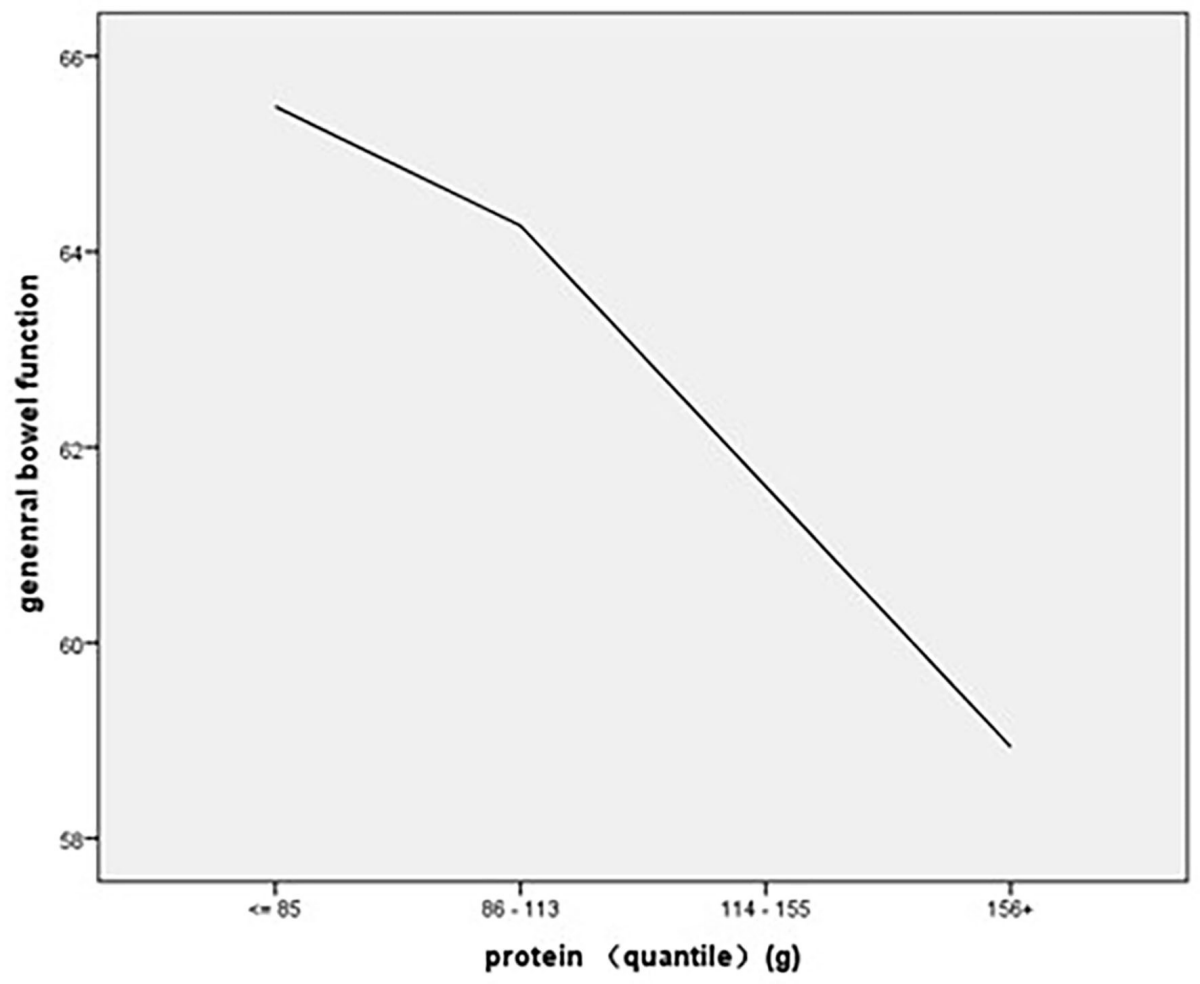
The association of stratified quintiles of consumption of protein with general bowel function.

As to the association of stratified quintiles of consumption of food components with bowel function, the lowest quantile of fruit intake (0–152 g/day) led to the best general bowel function (Figure 1), and the lowest quantile of protein intake of 0–85 g/day led to the best general bowel function (Figure 2).

#### Regression Analysis of Diet Consumption on the Experience of Frequent and Urgent Defecation

**Figure 3:**
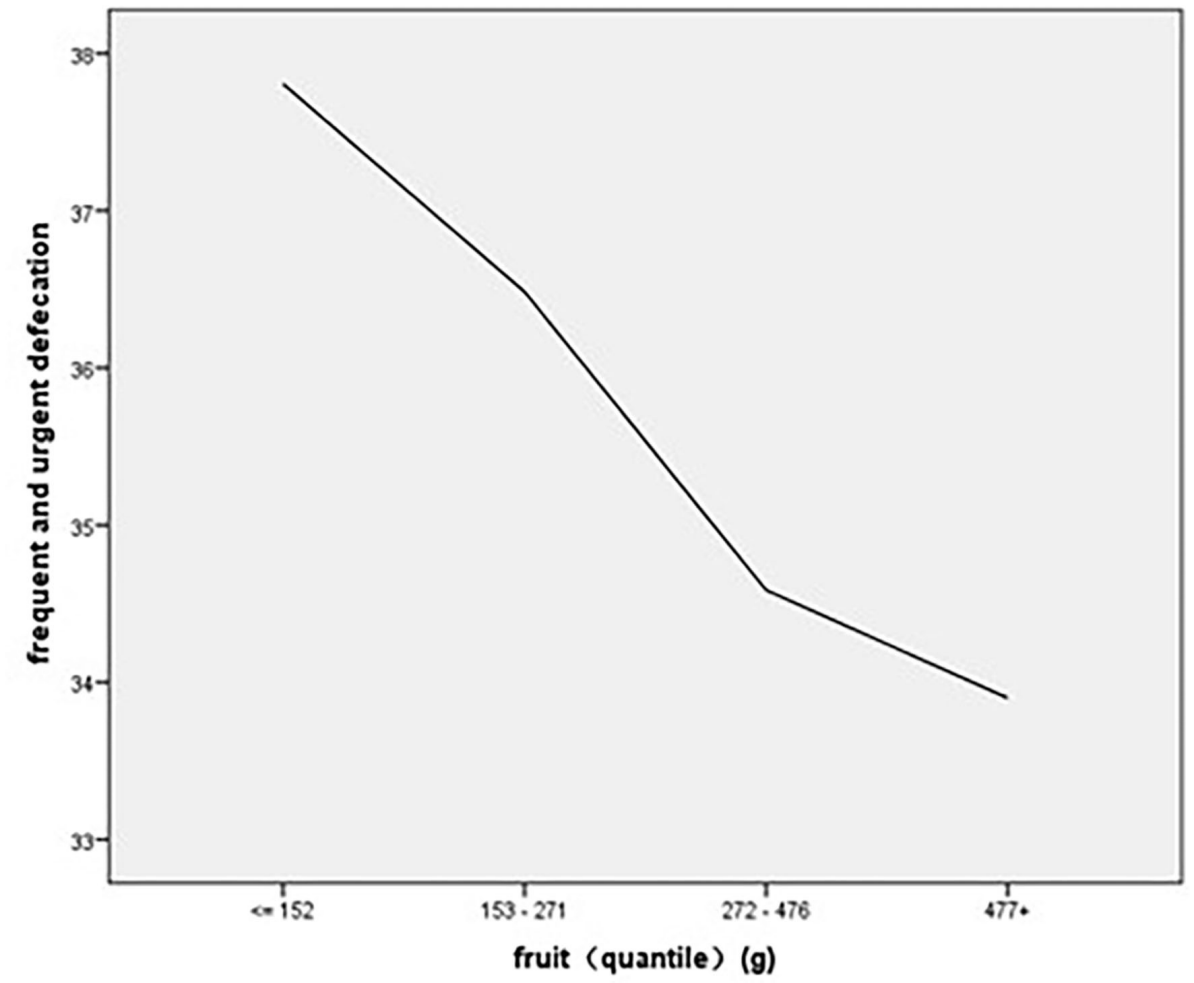
The association of stratified quintiles of consumption of fruits with frequent and urgent defecation.

**Figure 4:**
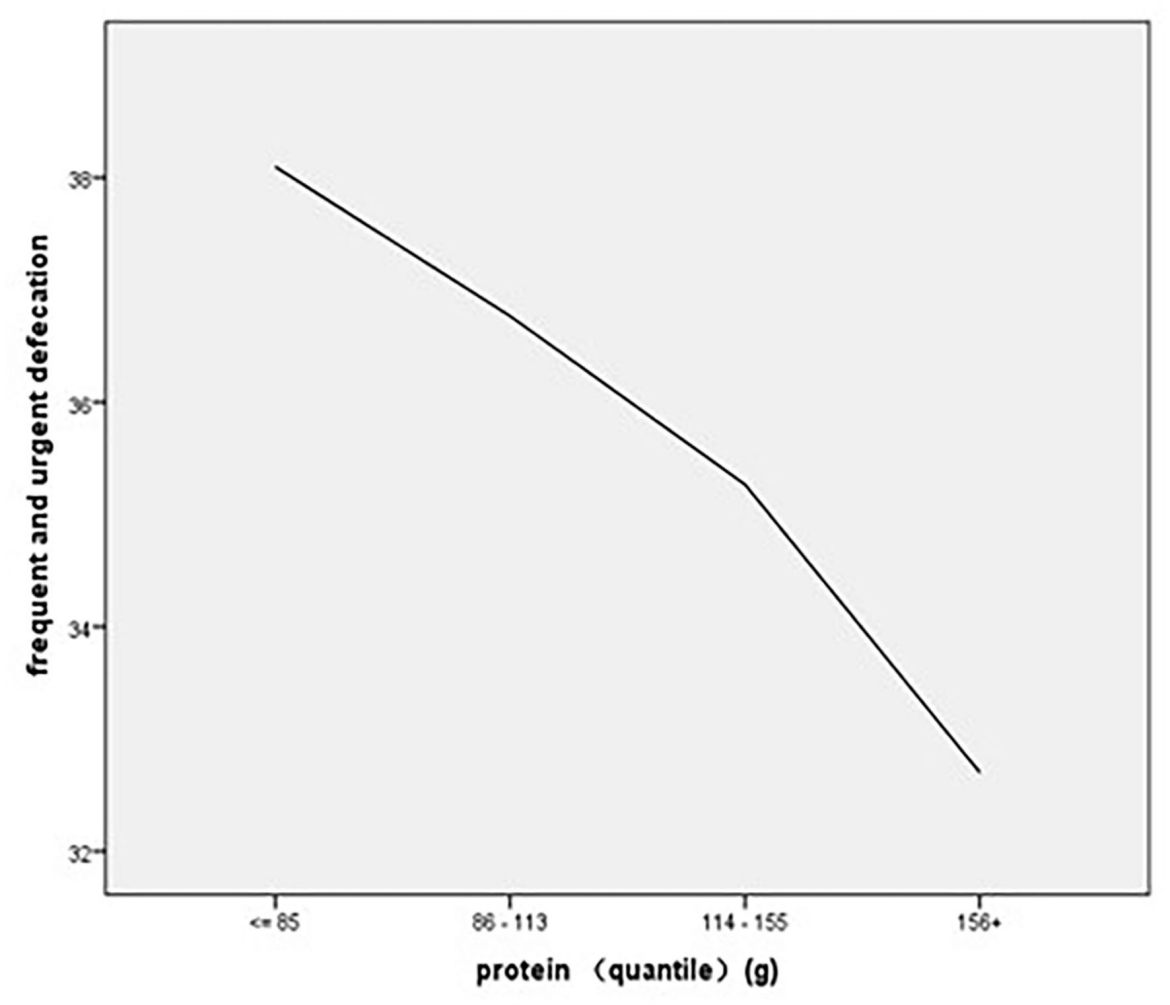
The association of stratified quintiles of consumption of protein with frequent and urgent defecation.

The regression results showed that protein consumption (β = −1.468, *p* < 0.01) and fruit consumption (β = −1.022, *p* < 0.01) had significant main effects on the frequent and urgent defecation experience, and the regression results showed an *R*^2^ of 24%, indicating that protein consumption and fruit consumption could explain 24% of the variance in the experience of frequent and urgent defecation (Table 5). As to the association of stratified quintiles of consumption of food components with bowel function, the lowest quantile of fruit intake (0–152 g/day) led to the least symptoms of frequent and urgent defecation (Figure 3), and the lowest quantile of protein intake of 0–85 g/day led to the least symptoms of frequent and urgent defecation (Figure 4).

**Table 5:** Regression analysis of diet consumption on experience of frequent and urgent defecation.

**Variable**	***B***	***Beta***	***t***	***p***
Constant	39.744	1.664	23.879	<0.001
Protein	−1.468	0.347	−4.232	<0.001
Fruit	−1.022	0.345	−2.964	0.004

*R^2^ = 0.259, adjusted R^2^ = 0.240, F = 13.598*.

#### Regression Analysis of Diet Consumption on the Effects of Diet on Defecation

**Figure 5:**
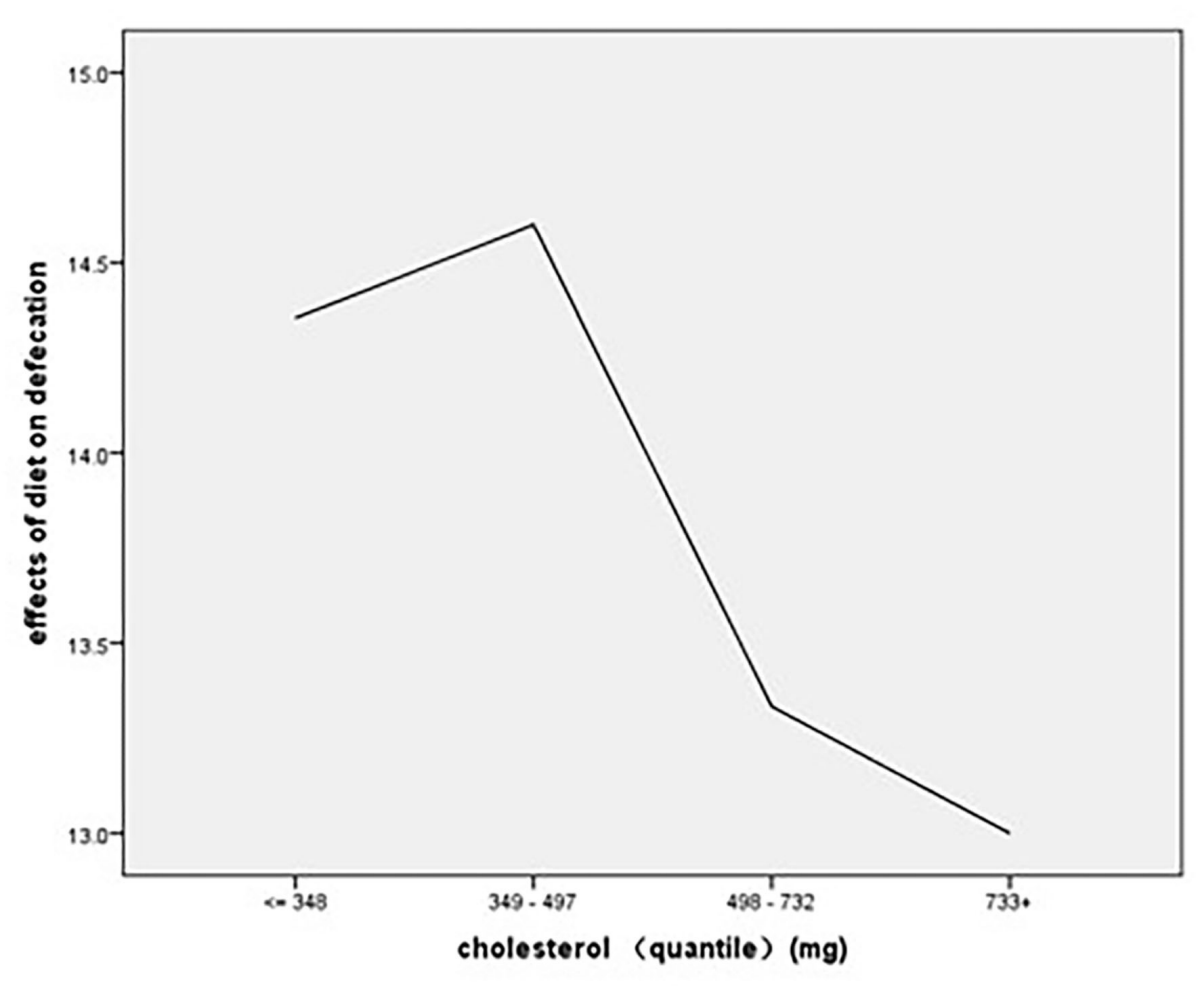
The association of stratified quintiles of consumption of cholesterol with effects of diet on defecation.

The regression results showed that cholesterol consumption (β = −0.541, *p* < 0.01) was a significant main factor affecting the effects of diet on the defecation score, and the regression results showed an *R*^2^ of 7.7%, indicating that cholesterol consumption could explain 7.7% of the variance in the effects of diet on defecation (Table 6). As to the association of stratified quantiles of consumption of food components with bowel function, the quantile of cholesterol intake of 349–497 mg/day led to the least symptoms caused by diet (Figure 5).

**Table 6:** Regression analysis of diet consumption on the effects of diet on defecation.

**Variable**	***B***	***Beta***	***t***	***P***
Constant	15.146	0.436	34.737	<0.001
Cholesterol	−0.541	0.160	−3.320	0.001

*R^2^ = 0.085, adjusted R^2^ = 0.077, F = 11.023*.

#### Regression Analysis of Diet Consumption on Abnormal Feelings of Defecation

The regression results showed that the interaction between cereals and milk products (β = 0.113, *p* < 0.01) and fruit consumption (β = −0.333, *p* < 0.05) had significant main effects on the abnormal feeling of defecation score, and the regression results showed an *R*^2^ of 8.1%, indicating that the interaction between cereals and milk products and fruit consumption could explain 8.1% of the variance in general bowel function (Table 7).

**Table 7:** Regression analysis of diet consumption on the abnormal feelings of defecation.

**Variable**	***B***	***Beta***	***t***	***p***
Constant	13.227		30.844	<0.001
Cereals*milk products	0.113	0.263	2.985	0.003
Fruits	−0.333	−0.146	−2.281	0.024

*R^2^ = 0.097, adjusted R^2^ = 0.081, F = 6.304*.

*The regression analysis have all been conducted after the adjustment of age, status of radiotherapy, distance between the margin of anal and tumor, consumption of energy*.

**Figure 6:**
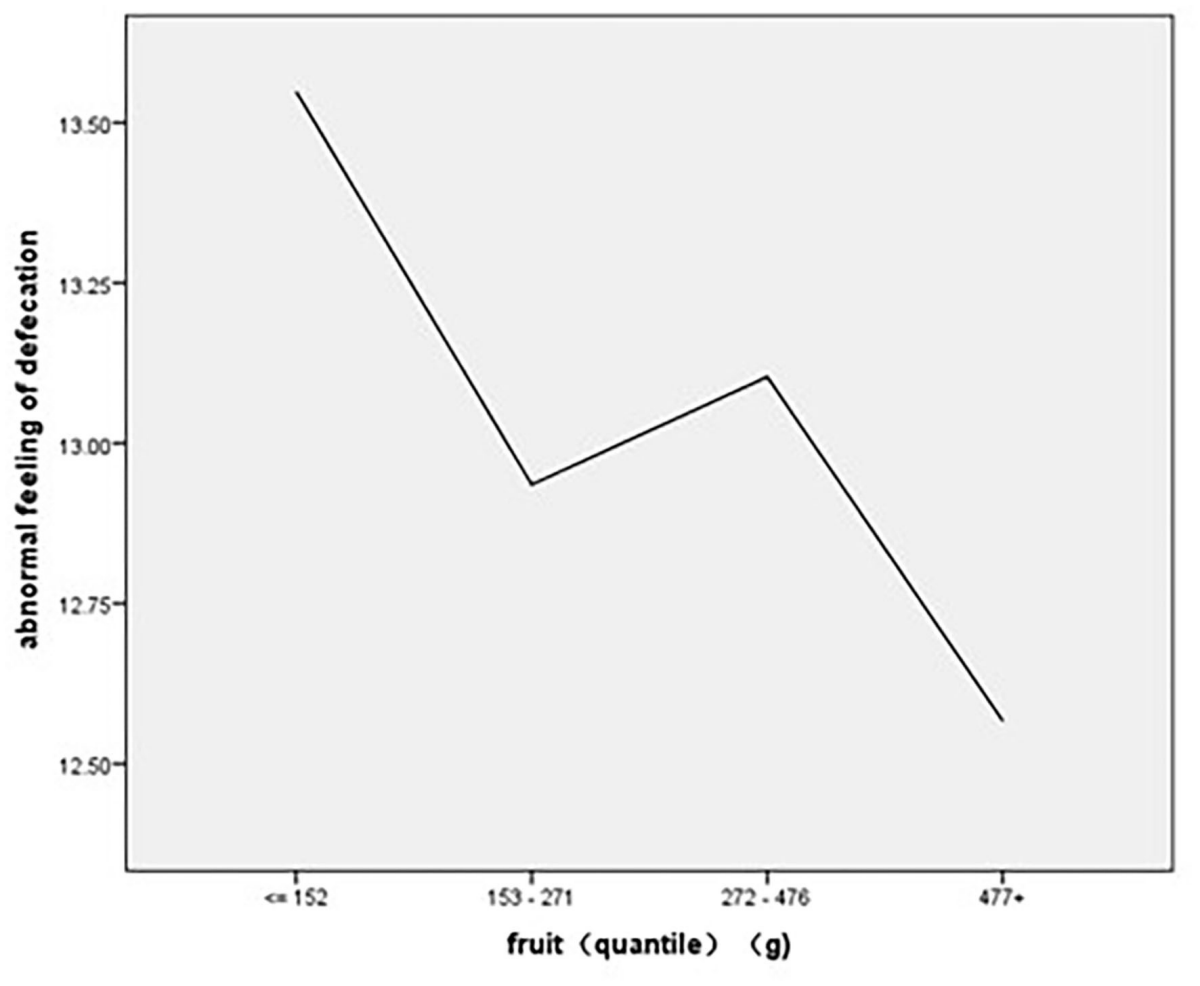
The association of stratified quintiles of consumption of fruits with abnormal feeling of defecation.

**Figure 7:**
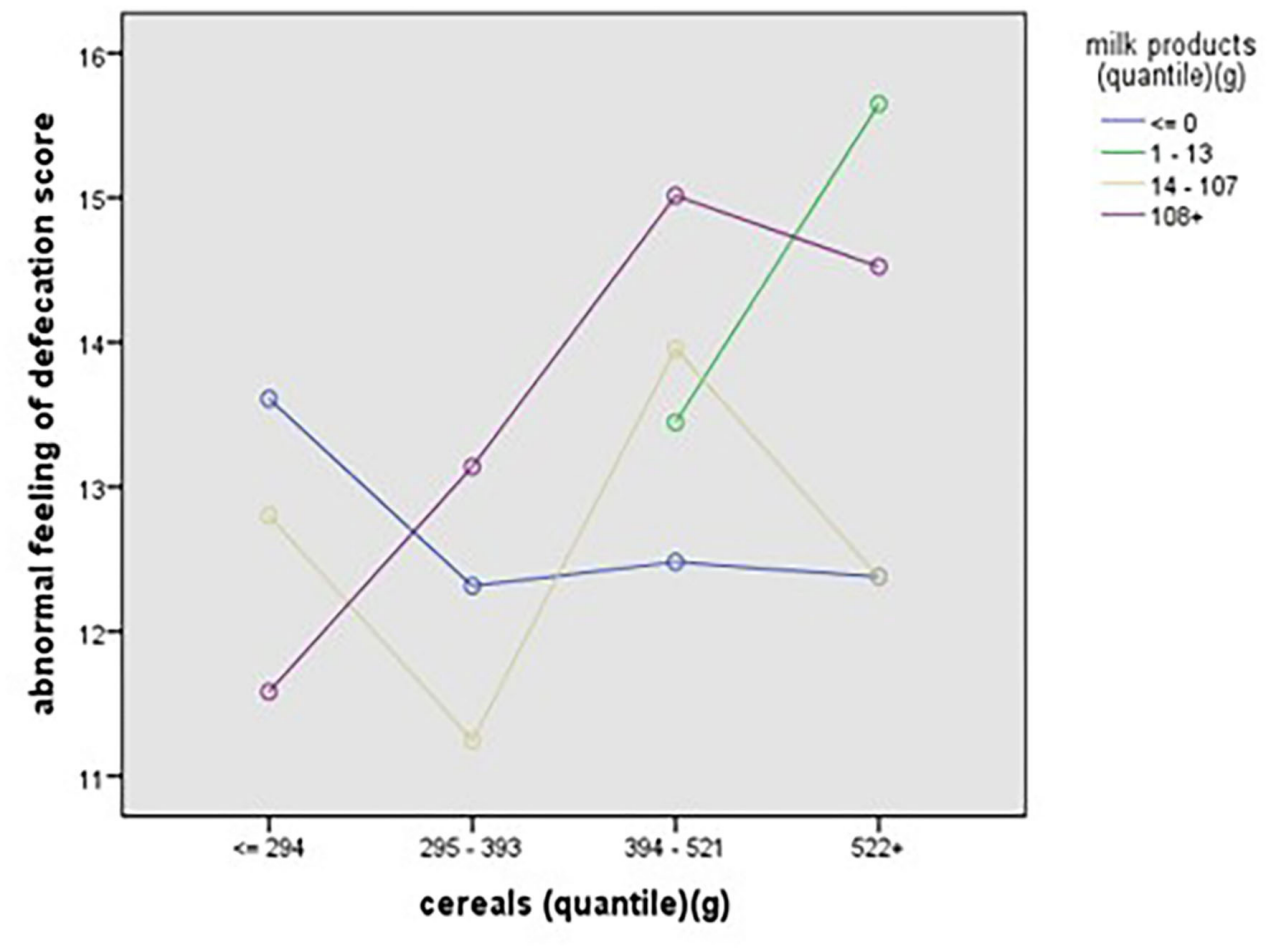
The association of interaction between cereals and milk products with abnormal feeling of defecation.

As to the association of stratified quintiles of consumption of food components with bowel function, the lowest quantile of fruit intake of 0–152 g/day led to the least symptoms of abnormal feeling of defecation (Figure 6). The association of interaction between cereals and milk products with abnormal feeling of defecation is shown in Figure 7.

## Discussion

Diet was considered as an important factor that had certain effects on bowel symptoms in past studies. The qualitative method was used most commonly in past studies in which patients' experience reflected by themselves was the main evidence to explore the relationship between diet and bowel symptoms (16, 19). In this study, the status of defecation dysfunction and the consumption of food components were assessed separately by specific questionnaires, and the direct relationship between the consumption of specific food components and bowel symptoms was first identified and analyzed among patients after sphincter-saving surgery.

In this study, the fat consumption of patients after sphincter-saving surgery did not show a significant relationship with frequent and urgent defecation, which is not consistent with the results in past studies. High fiber and low fat were the most common patterns of diet modification applied by patients, which was reported in past studies (15, 19, 20). The qualitative research conducted by Sun et al. showed that patients after sphincter-saving surgery reported that food rich in fat could aggravate diarrhea (15); another two studies focusing on the incontinence symptoms of patients reflected that greasy and fried foods were the most common types of food aggravating the burden of fecal incontinence (19, 20).

Protein was also shown in the multivariate analysis to be an important influential factor of the bowel function of patients. General bowel function and the symptom of frequent and urgent defecation would be significantly worse if patients took in more protein in a daily diet. However, the effect of protein consumption was mixed and inconsistent among different patients in the study of Crosswell et al. Among the 188 patients with symptoms of fecal continence, 8.6% tried to control fecal continence by reducing the amount of protein consumed in their diets, while 1.6% tried to control fecal continence by taking in more foods rich in protein. The definitive relationship between protein intake and bowel symptoms needs to be identified in future studies (20).

The multivariate analysis results showed that the consumption of fruit had a direct effect on bowel symptoms, including frequent and urgent defecation, abnormal feeling of defecation, and general bowel function. This study also revealed that a higher consumption of fruit led to more obvious bowel symptoms, especially more frequent and urgent defecation and more obvious abnormal feeling of defecation. This effect of fruits was consistent with several past studies conducted by Taylor et al. (16) and Crosswell et al. (20). Taylor et al. pointed out that the skins and peels of fruits that were rich in insoluble fiber resulted in defecation dysfunction by causing more frequent bowel motions. Crosswell et al. found that 55% of adults perceived that some foods worsened their fecal continence, and 11.2% of adults reflected that some fruits, such as raisins, had the effect of aggravating fecal continence. However, mixed effects of fruits on fecal continence were also found in this study, and 8.5% of adults tried to reduce or prevent fecal continence by eating apples, grapes, bananas, and raisins. These mixed effects were also reflected in another qualitative study conducted by Sun et al. (15). The interview results showed that some food categories, such as vegetables and fruits, were reported as being harmful by some survivors but helpful by others. The mixed effects might be caused by two reasons. The first reason was that the results emerged from patients' experiences and feelings, and the preference degree of fruits in daily lives was different among different patients, causing the discrepancy both in types of foods taken in by patients and in the feedback of the effects of the fruit group on bowel symptoms; for another reason, the component ratio of different fruits was not the same, so differences in the effects of fruit on defecation existed for different types of food.

In addition to fruit affecting bowel symptoms, the components of fruit are also considered to play an important role in patients' bowel symptoms. Although evidence of the relationship between different food components and bowel symptoms after sphincter-saving surgery is still scarce, fiber has been confirmed by many researchers to be an important factor affecting defecation, especially because it could directly affect fecal continence. The large prospective cohort study conducted by Staller et al. showed that compared with the rate of fecal continence of participants consuming 13 g of fiber per day, the rate of fecal continence among participants consuming 25 g of fiber per day decreased by 18% (12). The researcher pointed out that postoperative patients with colorectal cancer were also included in the whole sample, and this conclusion could be deducted to these populations. This relationship was also shown in a phenomenology study conducted by Hansen et al. (19), in which one-to-one interviews were separately conducted with 10 women who suffered from fecal continence, and the results showed that more than half of these women mentioned that high-fiber foods or fiber supplements were helpful in controlling fecal incontinence. On the other hand, some researchers reported that a lower amount of fiber could lead to fewer bowel symptoms, especially among patients after pelvic radiotherapy (21). The different effects caused by fiber were considered to be related to the type of fiber ingested. The study of Bliss et al. of community-living adults with fecal incontinence showed that supplementation with soluble dietary fiber improves the water-holding capacity of stool solids, the consistency of stools, and fecal incontinence (22). In contrast, insoluble fiber derived from foods such as fruit peels may exacerbate diarrhea and bloating, which was also reported in the investigation of Taylor et al. (16). In this study, the relationship between fiber and bowel symptoms was not significant, which may be because we only considered the total consumption of fiber, and these two different types of fiber with different functions were not assessed and analyzed separately. More studies aiming to further analyze the specific effects of the two types of fiber on the defecation of patients after sphincter-saving surgery are needed in the future.

The consumption of cholesterol was also found to have a significant effect on effects of diet on defecation. As the consumption of cholesterol increased, the effects of diet on defecation became more obvious. Cholesterol mainly existed in foods of animal origin like beef, pork, egg yolk, milk products, and so on. There was limited evidence related to the relationship between cholesterol and bowel function among rectal cancer patients after sphincter-saving surgery. According to previous research, some patients chose to control bowel symptoms after surgery by cutting down the consumption of meat (15, 16). The research of Sun et al. indicated that milk products were reflected to have the possibility of aggravating bowel symptoms and 7.9% of patients chose to avoid taking milk products after sphincter surgery (15). More research is needed in the future to identify the specific relationship between the consumption of foods rich in cholesterol and bowel symptoms after sphincter-saving surgery.

Diet modification was chosen as the first choice for patients to manage bowel symptoms, and trial and error is the main method applied by patients, leading to some problems and barriers in the process of modification. In the qualitative study of Hansen et al. (19), diet modification was found to be uncontrolled and not evidenced, resulting in the possible consequences of an imbalanced diet, micronutrient deficiency, and protein-calorie malnutrition. This study also reported the lack of therapeutic guidance regarding diet modification among patients with fecal incontinence. This was consistent with the results of the study of Bours et al. (23), which showed that among 1,458 colorectal patients, only 17% had been given dietary information support. In study of Nikoletti et al. (13), 55% of patients after sphincter-saving surgery reflected that diet-related information was in greatest need, and clearly, written information about the possible effects of diet and diet supplements along with guidelines on how to identify problem foods was also needed among rectal patients after surgery. Without proper diet modification, patients reported that their quality of life was worse (15). Therefore, nursing programs aimed at providing scientific guidance on dietary modification need to be established in future studies.

The study has several limitations. First, it was a cross-sectional study that made it difficult to attribute causality to the relationships among these factors, and it cannot provide information on the dynamic changes in food consumption and bowel symptoms of patients after sphincter-saving surgery. Second, the sample size was relatively small, and patients with rectal cancer were all recruited from only one tertiary hospital, which might limit the generalization of the findings. Third, the effects of medical data and other social–psychological factors on food consumption and bowel symptoms were not fully considered in this study. In the future, multicenter longitudinal studies that include comprehensive relevant factors should be conducted to verify our results.

## Conclusions

This study was the first to explore the relationships among the consumption of different food components and bowel symptoms after sphincter-saving surgery. In this study, the consumption of fruit, cholesterol, and protein and the interaction of cereals and milk products were linked with general bowel symptoms. These findings provide evidence for medical staff to further develop scientific dietary education programs to relieve bowel symptoms in the future. Considering the mixed effects of certain foods on bowel symptoms in other studies, more research is needed to explore the causal relationship among specific food components and bowel symptoms; meanwhile, the mechanisms of the effects of different food components on bowel symptoms among patients after sphincter-saving surgery need to be studied and identified in future studies.

## Declaration

The lead author affirms that this manuscript is an honest, accurate, and transparent account of the study being reported. The lead author affirms that no important aspects of the study have been omitted and that any discrepancies from the study as planned and the study was approved by the Ethics Committee of School of Nursing of Fudan University.

## Data Availability Statement

The data that support the findings of this study are available from the corresponding author upon reasonable request.

## Ethics Statement

This study was approved by Ethics Committee of Zhongshan Hospital, Fudan University (No: B2019-318R). The patients/participants provided their written informed consent to participate in this study.

## Author Contributions

HX was responsible for the organization of the manuscript and the study design. WL was responsible for the majority of the draft writing. JX and YZ contributed the data collection in this manuscript. HL was responsible for the study conception and made critical modification and revisions for the draft. All authors contributed to the article and approved the submitted version.

## Conflict of Interest

The authors declare that the research was conducted in the absence of any commercial or financial relationships that could be construed as a potential conflict of interest.
